# Supplementary material for: Effects of Waterlogging on Soybean Rhizosphere Bacterial Community Using V4, LoopSeq, and PacBio 16S rRNA Sequence
Source: Microbiol Spectr. 2022 Feb 16;10(1):e02011-21. doi: 10.1128/spectrum.02011-21 (PMC8849089; doi:10.1128/spectrum.02011-21)
Supplement: SUPPLEMENTAL FILE 1 — Supplemental material. Download SPECTRUM02011-21_Supp_1_seq10.pdf, PDF file, 0.6 MB [file spectrum02011-21_supp_1_seq10.pdf]

Effects of waterlogging on soybean rhizosphere bacterial community using V4, LoopSeq and PacBio 16S rRNA sequence

Taobing Yu[a,b], Lang Cheng[a,b], Qi Liu[a,b], Shasha Wang[a,b], Yuan Zhou[c], Hongbin Zhong[c], Meifang Tang[c], Hai Nian[a,b,*], Tengxiang Lian[a,b,*]

[a]*The State Key Laboratory for Conservation and Utilization of Subtropical Agro-bioresources, South China Agricultural University, Guangzhou 510642, Guangdong, People's Republic of China*

[b]*The Key Laboratory of Plant Molecular Breeding of Guangdong Province, College of Agriculture, South China Agricultural University, Guangzhou 510642, Guangdong, People's Republic of China*

[c]*BGI Genomics, BGI-Shenzhen, Shenzhen 518083, China*

[*] **Corresponding author1:** Tengxiang Lian

Corresponding address: No.483 Wushan Road, Guangzhou, Guangdong, 510642, China.

Tel: +86 02085288024

Fax: +86 02085288024

E-mail address: liantx@scau.edu.cn

[*] **Corresponding author2:** Hai Nian

Corresponding address: No.483 Wushan Road, Guangzhou, Guangdong, 510642, China.

Tel: +86 02085288024

Fax: +86 02085288024

mail address: hnian@scau.edu.cn

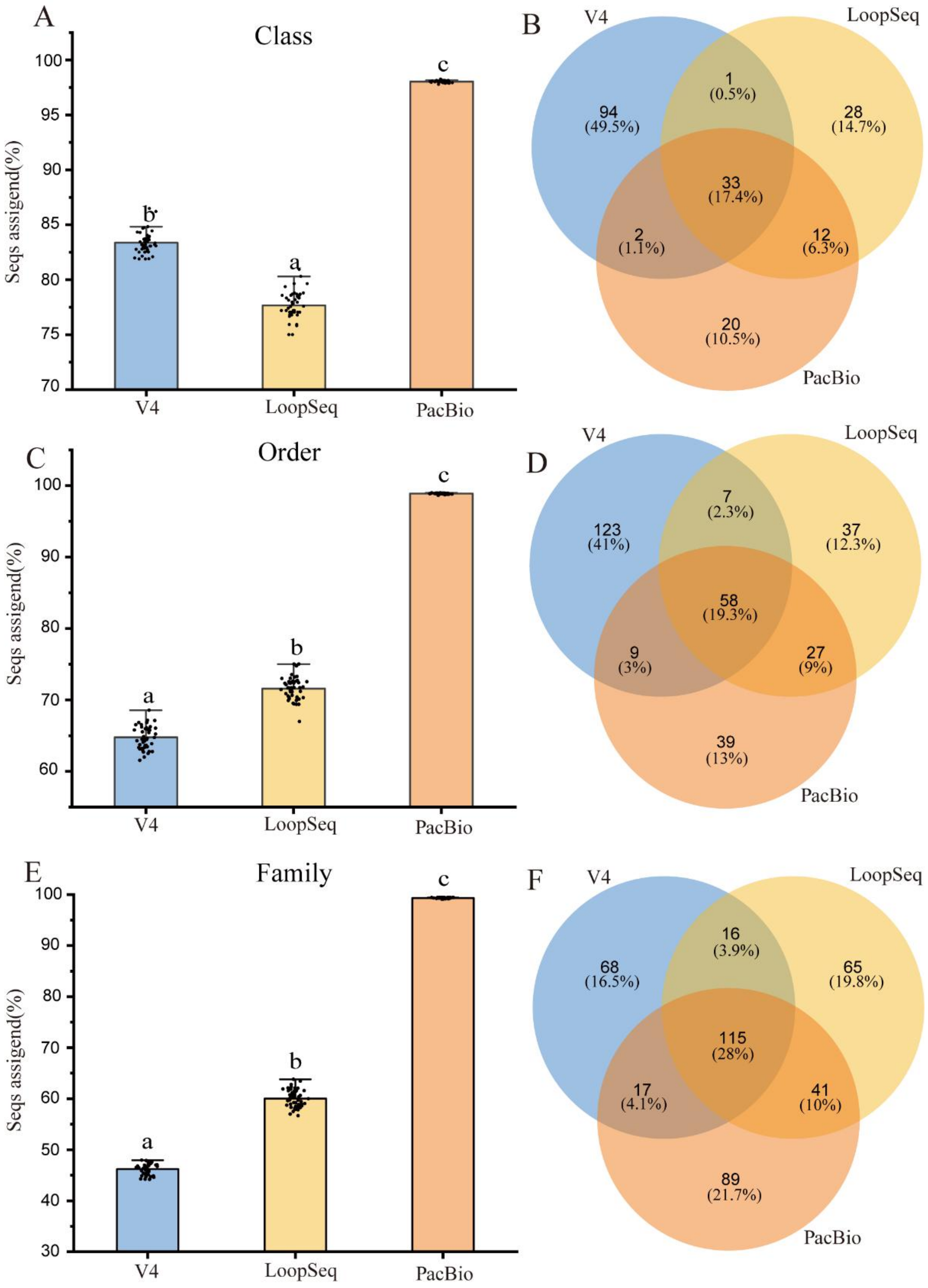

Fig. S1. Taxonomy profiles in different sequencing methods' datasets. The proportion of annotation sequences from the V4 (n =48, blue), LoopSeq (n= 48, yellow), and PacBio (n =48, orange) datasets was determined by comparing the sequence with the SILVA database, respectively, and are represented at the (A) class, (C) order, and (E) family levels. Venn diagram showing the numbers of unique and

shared (B) class, (D) order, and (F) family among the three sequencing methods. Blue denotes V4, yellow denotes LoopSeq, and orange denotes PacBio.

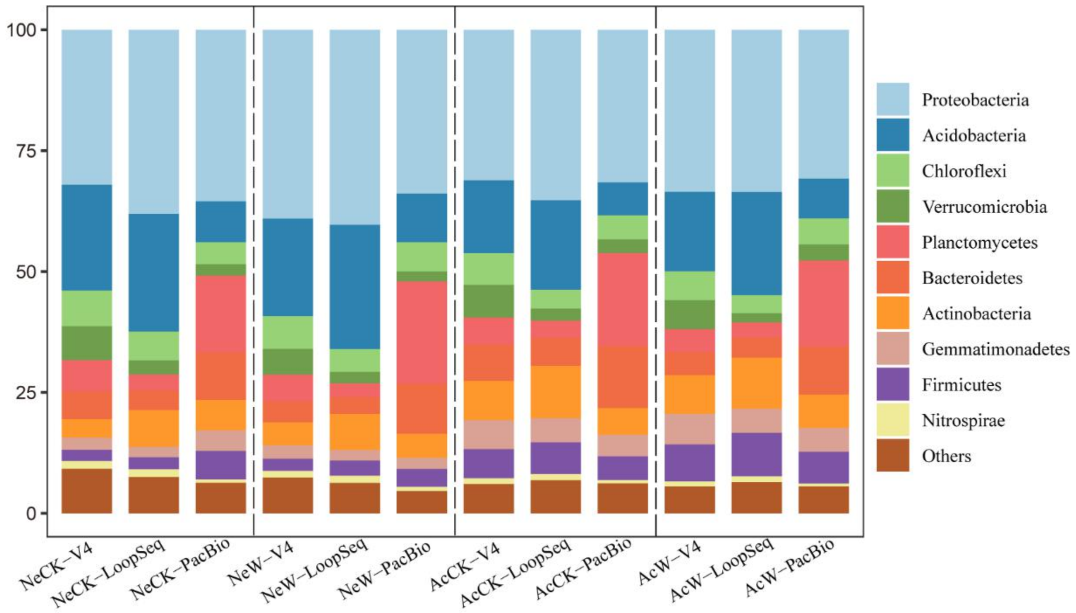

Fig. S2. The relative abundances of the bacterial phyla.

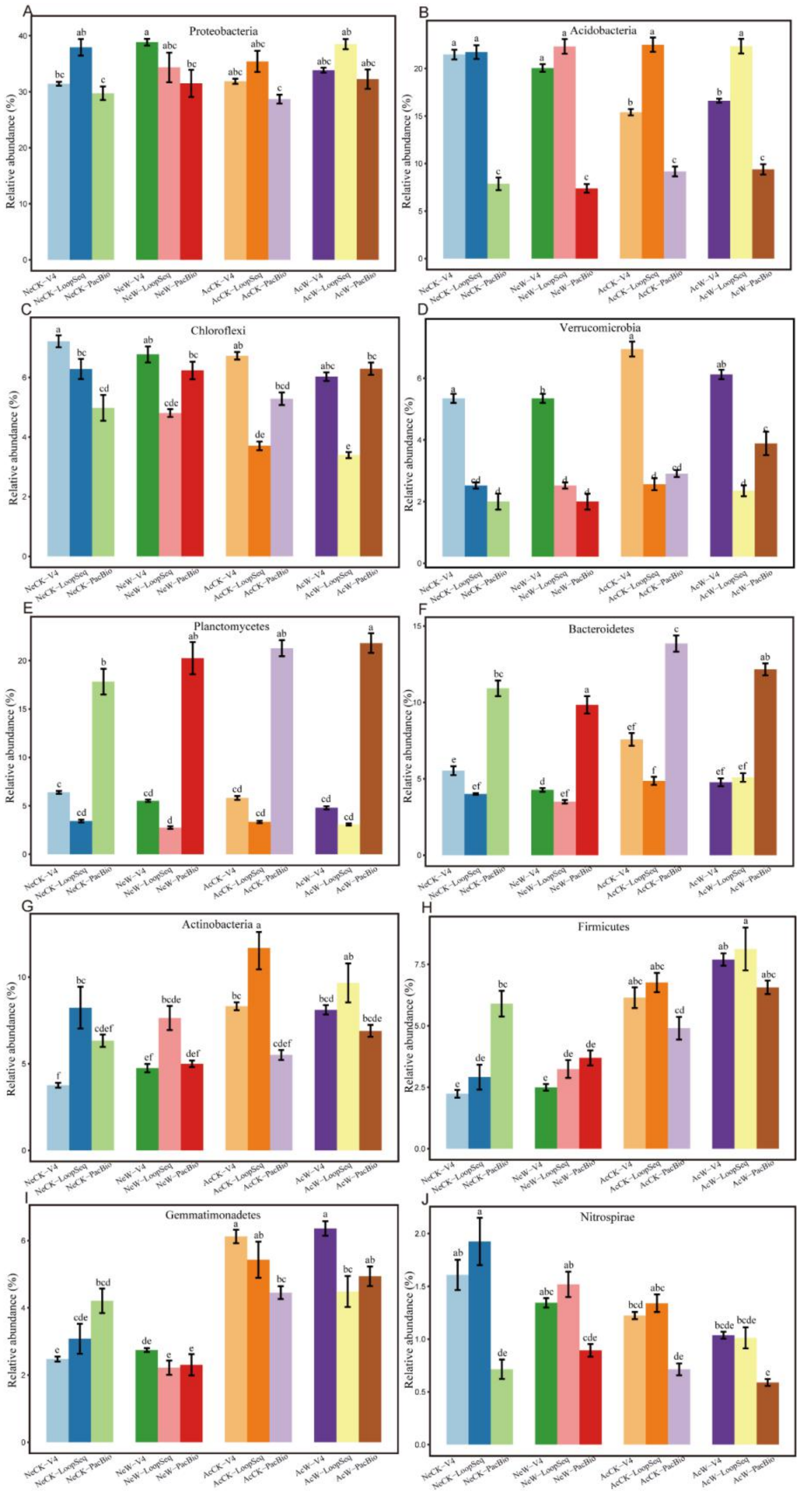

Fig. S3. Comparison of relative abundance of bacteria phylum with or without waterlogging in different soil types by different sequencing methods. Different colors denote different treatments. Different letters indicate significant differences (*P* < 0.05, one-way ANOVA, n=12).

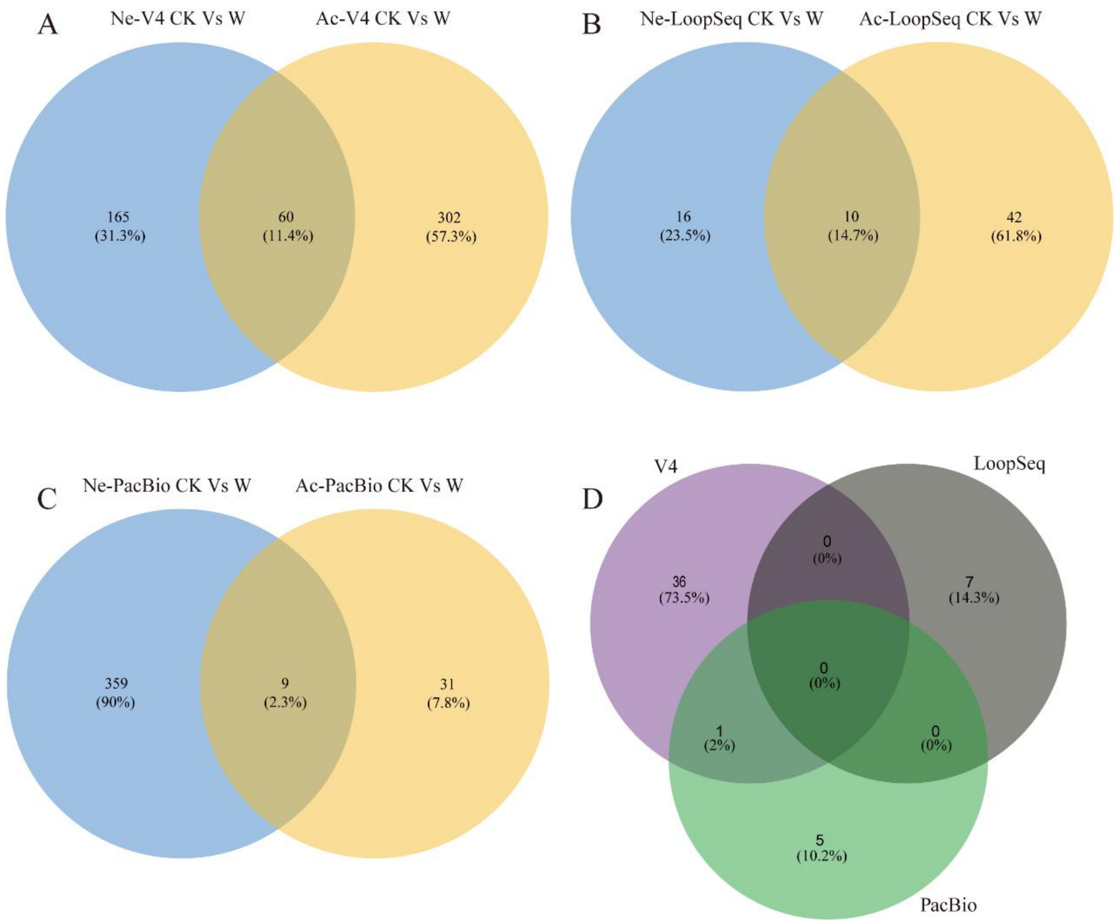

Fig. S4. (ABC)Venn diagram shows the unique and shared number of different OTUs with and without waterlogging on the two soil types with three sequencing methods. (D)The unique and shared differences between waterlogging and without waterlogging OTU in neutral soil and acidic soil were classified to genus level by V4, LoopSeq, and Pacbio sequencing methods. Blue denotes OTU with a significant difference between waterlogging and without waterlogging in neutral soil; yellow denotes OTU with significant difference between waterlogging and without waterlogging in acidic soil; violet denotes V4, grey denote LoopSeq, green denotes PacBio

Table S1 Waterlogging, soil type, and their interactive effects on soil physicochemical properties.

Soil physicochemical properties	AcCK	AcW	NeCK	NeW	Soil type	Waterlogging	Soil type * Waterlogging	LSD
SOC (g/kg)	29.84±1.87	30.27±4.37	41.04±3.95	42.73±4.88	**<0.001**	0.582	0.357	3.24
pH	5.26±0.27	5.38±0.38	7.1±0.28	7.11±0.32	**<0.001**	0.478	0.573	0.2575
TN (g/kg)	1.52±0.09	1.53±0.09	1.99±0.16	2.05±0.2	**<0.001**	0.459	0.577	0.116
TP (g/kg)	0.42±0.1	0.45±0.08	1.22±0.19	1.31±0.29	**<0.001**	0.256	0.544	0.1529
AP (mg/kg)	11.63±0.78	13.78±1.63	34.81±2.9	38.14±3.34	**<0.001**	**<0.001**	0.399	1.965
AK (mg/kg)	34.09±1.99	34.35±5.97	78.82±17.92	87.57±18.62	**<0.001**	0.274	0.247	10.94
NH_4^+ (mg/kg)	7.97±2.34	12.44±1.46	27.99±4.18	35.59±4.57	**<0.001**	**<0.001**	0.117	2.789
NO_3^- (mg/kg)	13.92±3.78	23.77±2.97	33.66±11.87	66.45±8.4	**<0.001**	**<0.001**	**<0.001**	6.3

P values less than 0.05 were indicated in bold letters.

Table S2 Waterlogging, soil type, sequencing method and their interactive effects the diversity and classification resolution.

	Diversity		Classification resolution					
	Chao1	Shannon	Phylum	Class	Order	Family	Genus	Species
AcCK-V4	640.77±1.71	5.78±0.07	97.41±0.08	83.11±0.92	63.55±0.96	45.65±0.75	45.13±0.76	14.26±0.59
AcW-V4	640.75±1.51	5.7±0.05	97.44±0.11	83.31±0.72	63.61±0.94	45.29±0.93	44.95±0.78	14.28±0.45
NeCK-V4	496.04±15.18	5.11±0.05	97.13±0.11	83.63±1.19	65.45±1.46	47.22±0.51	42.89±0.72	15.4±0.46
NeW-V4	477.62±12.97	4.98±0.07	97.19±0.15	83.43±1.12	66.42±0.51	47.24±0.47	41.82±0.6	15.4±0.49
AcCK-LoopSeq	270.08±44.53	5.25±0.13	95.84±0.31	78.2±1.31	71.09±1.95	61.61±0.98	87.53±0.67	28.26±2.85
AcW-LoopSeq	264.17±54.97	5.31±0.2	95.55±0.33	77.37±0.89	72.29±1.26	59.52±1.49	86.63±0.8	32.11±4.87
NeCK-LoopSeq	172.42±115.69	4.59±0.85	95.53±0.46	77.77±1.35	71.55±1.6	60.04±1.97	87.18±1.01	27.82±4.51
NeW-LoopSeq	264.58±26.63	5.3±0.1	95.39±0.29	77.35±1.34	71.43±1.31	59.98±1.19	86.91±0.48	34.13±1.98
AcCK-PacBio	242.32±22.59	4.85±0.24	95.34±0.26	98.03±0.08	98.87±0.07	99.31±0.09	97.51±0.12	99.7±0.09
AcW-PacBio	267.77±7.5	5.16±0.07	95.26±0.31	98.06±0.13	98.89±0.11	99.34±7.66	97.39±0.22	99.73±0.09
NeCK-PacBio	247.28±32.2	4.87±0.23	95.1±0.12	98.02±0.06	98.86±0.05	99.34±0.11	97.29±0.21	99.73±0.04
NeW-PacBio	269.18±6.22	5.13±0.1	95.23±0.28	98.05±0.07	98.89±0.05	99.35±0.04	97.25±0.13	99.75±0.04
Sequencing method	**<0.001**	**<0.001**	**<0.001**	**<0.001**	**<0.001**	**<0.001**	**<0.001**	**<0.001**
Soil type	**<0.001**	**<0.001**	0.26	0.202	**0.048**	**<0.001**	**<0.001**	**<0.001**
Waterlogging	**0.007**	**<0.001**	**<0.001**	0.862	**<0.001**	**<0.001**	**<0.001**	0.077
Sequencing method * Soil type	**<0.001**	**<0.001**	**0.025**	0.148	0.442	**<0.001**	0.055	**<0.001**
Soil type * Waterlogging	0.072	**0.042**	0.138	0.997	0.7	**<0.001**	0.773	0.269
Sequencing method * Waterlogging	**0.01**	**<0.001**	0.408	0.347	**<0.001**	**<0.001**	**<0.001**	0.452
Sequencing method * Soil type * Waterlogging	**0.001**	**0.003**	0.678	0.569	**0.039**	**<0.001**	**0.01**	0.28
LSD	33.9	0.2253	0.2096	0.7457	0.8684	2.069	0.4964	1.761

P values less than 0.05 were indicated in bold letters.

Table S3 The core rhizosphere microorganisms of different sequencing methods.

	OTU ID	AcCK	AcW	NeCK	NeW	Phylum	Class	Order	Family	Geuns	Species
V4	OTU14	2.01±0.28	1.73±0.18	1.02±0.17	0.88±0.1	Proteobacteria	Betaproteobacteria	SC-I-84	unclassified_SC-I-84	unclassified_SC-I-84	unclassified_SC-I-84
	OTU18	0.91±0.12	0.98±0.13	1.54±0.22	1.25±0.12	Acidobacteria	Acidobacteriia	Acidobacteriales	Koribacteraceae	unclassified_Koribacteraceae	unclassified_Koribacteraceae
	OTU19	0.8±0.08	0.88±0.16	1.91±0.33	1.46±0.11	Acidobacteria	Acidobacteriia	Acidobacteriales	Koribacteraceae	unclassified_Koribacteraceae	unclassified_Koribacteraceae
	OTU63	0.76±0.13	0.92±0.15	0.64±0.12	0.84±0.09	Proteobacteria	Betaproteobacteria	Ellin6067	unclassified_Ellin6067	unclassified_Ellin6067	unclassified_Ellin6067
	OTU13	1.94±0.3	1.75±0.2	0.96±0.27	0.59±0.07	Verrucomicrobia	Spartobacteria	Chthoniobacterales	Chthoniobacteraceae	DA101	unclassified_DA101
	OTU20	1.89±0.46	1.47±0.53	0.56±0.14	0.71±0.08	Proteobacteria	Betaproteobacteria	Burkholderiales	Comamonadaceae	unclassified_Comamonadaceae	unclassified_Comamonadaceae
	OTU17	0.73±0.09	0.53±0.1	0.9±0.13	0.68±0.1	Nitrospirae	Nitrospira	Nitrospirales	Nitrospiraceae	Nitrospira	unclassified_Nitrospira
	OTU71	0.6±0.11	0.53±0.07	0.71±0.16	0.75±0.13	Acidobacteria	Acidobacteria-6	iii1-15	unclassified_iii1-15	unclassified_iii1-15	unclassified_iii1-15
	OTU1	0.1±0.03	3.88±1.5	0.6±0.19	3.82±1.07	Proteobacteria	Deltaproteobacteria	Desulfuromonadales	Geobacteraceae	Geobacter	unclassified_Geobacter
	OTU79	0.01±0.01	0±0	0.24±0.09	0.14±0.03	Bacteroidetes	Cytophagia	Cytophagales	Cytophagaceae	unclassified_Cytophagaceae	unclassified_Cytophagaceae
	OTU951	0.73±0.16	0.88±0.13	0.2±0.05	0.35±0.05	Proteobacteria	Betaproteobacteria	Burkholderiales	Comamonadaceae	Variovorax	unclassified_Variovorax
	OTU11	0.97±0.12	0.87±0.17	0.28±0.07	0.26±0.04	Gemmatimonadetes	Gemmatimonadetes	N1423WL	unclassified_N1423WL	unclassified_N1423WL	unclassified_N1423WL
	OTU8	1.75±0.17	1.6±0.17	0.31±0.06	0.34±0.06	Proteobacteria	Alphaproteobacteria	Sphingomonadales	Sphingomonadaceae	Kaistobacter	unclassified_Kaistobacter
	OTU35	1.02±0.15	0.92±0.13	0.47±0.09	0.51±0.08	Acidobacteria	Acidobacteria-6	iii1-15	unclassified_iii1-15	unclassified_iii1-15	unclassified_iii1-15
	OTU4	2.42±0.55	2.42±0.5	0.44±0.11	0.42±0.13	Firmicutes	Bacilli	Bacillales	Bacillaceae	Bacillus	Bacillus_fumarioli
	OTU28	0.34±0.07	0.32±0.08	1.34±0.21	1.69±0.22	Proteobacteria	Betaproteobacteria	MND1	unclassified_MND1	unclassified_MND1	unclassified_MND1
	OTU34	0.2±0.03	0.18±0.03	1.41±0.14	1.16±0.11	Acidobacteria	Acidobacteriia	Acidobacteriales	Koribacteraceae	unclassified_Koribacteraceae	unclassified_Koribacteraceae
	OTU5	0.35±0.05	0.31±0.06	1.8±0.42	1.79±0.35	Proteobacteria	Alphaproteobacteria	Rhizobiales	Hyphomicrobiaceae	Rhodoplanes	unclassified_Rhodoplanes
LoopSeq	OTU4745	0.93±0.35	0.87±0.17	0.24±0.33	0.27±0.14	Firmicutes	Bacilli	Bacillales	Paenibacillaceae	Cohnella	Ambiguous_taxa
	OTU4746	0±0	0±0	1.23±1.05	0.95±0.49	Firmicutes	Bacilli	Bacillales	Bacillaceae	Bacillus	Ambiguous_taxa
	OTU1673	0.11±0.07	0.07±0.06	2.01±2.22	0.73±0.31	Actinobacteria	Actinobacteria	Micromonosporales	Micromonosporaceae	Dactylosporangium	Ambiguous_taxa
	OTU4531	0±0	0.75±0.32	0.07±0.08	1.22±0.64	Actinobacteria	Actinobacteria	Micrococcales	Intrasporangiaceae	Oryzihumus	Oryzihumus leptocrescens
PacBio	OTU5	0.94±0.22	0.71±0.29	0.75±0.44	0.18±0.26	Proteobacteria	Betaproteobacteria	Burkholderiales	Comamonadaceae	Ramlibacter	Ramlibacter_ginsenosidimutans
	OTU10	0.51±0.17	0.7±0.13	0.51±0.23	0.16±0.16	Proteobacteria	Betaproteobacteria	Nitrosomonadales	unclassified	unclassified	unclassified
	OTU11	0.68±0.19	0.62±0.21	0.56±0.28	0.15±0.2	Proteobacteria	Betaproteobacteria	Burkholderiales	Burkholderiaceae	unclassified	unclassified
	OTU248	0.62±0.18	0.79±0.38	0.43±0.28	0.07±0.05	Proteobacteria	Alphaproteobacteria	Sphingomonadales	Sphingomonadaceae	Sphingomonas	Sphingomonas_daechungensis
	OTU8	0.42±0.12	0.54±0.09	0.48±0.15	0.29±0.1	Bacteroidetes	Chitinophagia	Chitinophagales	Chitinophagaceae	unclassified	unclassified
	OTU3	0.69±0.16	0.73±0.17	0.56±0.3	0.07±0.18	Bacteroidetes	Chitinophagia	Chitinophagales	Chitinophagaceae	Flavisolibacter	Flavisolibacter_ginsengisoli
	OTU80	0.66±0.15	1.17±0.38	0.66±0.36	0.18±0.36	Bacteroidetes	Chitinophagia	Chitinophagales	Chitinophagaceae	Flavisolibacter	Flavisolibacter_ginsengisoli
	OTU22	0.46±0.15	0.39±0.1	0.45±0.22	0.36±0.12	Nitrospirae	Nitrospira	Nitrospirales	Nitrospiraceae	Nitrospira	Nitrospira_moscoviensis
	OTU2	0.4±0.09	0.48±0.23	0.37±0.21	0.2±0.12	Gemmatimonadetes	Gemmatimonadetes	Gemmatimonadales	Gemmatimonadaceae	Gemmatimonas	Gemmatimonas_phototrophica
	OTU196	0.42±0.11	0.42±0.11	0.37±0.22	0.02±0.06	Planctomycetes	Planctomycetia	Planctomycetales	Isosphaeraceae	Aquisphaera	Aquisphaera_giovannonii

Table S4 The enrich and deplete OTUs in waterlogging soil in the main modules of the three sequencing methods

	OTU ID	CK	W	Phylum	Class	Order	Family	Geuns	Species
V4-W-module2（significantly enriched）	OTU2501	0±0.01	0±0	Bacteroidetes	Cytophagia	Cytophagales	Cytophagaceae	unclassified_Cytophagaceae	unclassified_Cytophagaceae
	OTU78	0.05±0.08	0.02±0.02	Bacteroidetes	Sphingobacteriia	Sphingobacteriales	Sphingobacteriaceae	Mucilaginibacter	Mucilaginibacter_ximonensis
	OTU206	0.26±0.3	0.1±0.1	Bacteroidetes	Saprospirae	Saprospirales	Chitinophagaceae	Niastella	unclassified_Niastella
	OTU1454	0.03±0.03	0.01±0.01	Chloroflexi	C0119	unclassified_C0119	unclassified_C0119	unclassified_C0119	unclassified_C0119
	OTU546	0.04±0.03	0.01±0.01	Chloroflexi	C0119	unclassified_C0119	unclassified_C0119	unclassified_C0119	unclassified_C0119
	OTU854	0.02±0.02	0.01±0.01	Planctomycetes	vadinHA49	DH61	unclassified_DH61	unclassified_DH61	unclassified_DH61
	OTU138	0.08±0.08	0.04±0.03	Proteobacteria	Betaproteobacteria	Methylophilales	Methylophilaceae	Methylobacillus	unclassified_Methylobacillus
	OTU1898	0.01±0.01	0±0	Proteobacteria	Deltaproteobacteria	MIZ46	unclassified_MIZ46	unclassified_MIZ46	unclassified_MIZ46
	OTU1744	0.01±0.01	0±0	TM7	TM7-1	unclassified_TM7-1	unclassified_TM7-1	unclassified_TM7-1	unclassified_TM7-1
	OTU2616	0±0.01	0±0	unclassified	unclassified	unclassified	unclassified	unclassified	unclassified
	OTU3911	0±0	0±0	unclassified	unclassified	unclassified	unclassified	unclassified	unclassified
	OTU7466	0±0	0±0	unclassified	unclassified	unclassified	unclassified	unclassified	unclassified
	OTU1954	0.01±0.01	0.01±0.01	Verrucomicrobia	Spartobacteria	Chthoniobacterales	Chthoniobacteraceae	OR-59	unclassified_OR-59
V4-W-module3（significantly enriched）	OTU612	0.03±0.03	0.01±0.01	Armatimonadetes	unclassified_Armatimonadetes	unclassified_Armatimonadetes	unclassified_Armatimonadetes	unclassified_Armatimonadetes	unclassified_Armatimonadetes
	OTU2464	0.01±0.02	0±0	Bacteroidetes	Flavobacteriia	Flavobacteriales	Flavobacteriaceae	Flavobacterium	Flavobacterium_succinicans
	OTU407	0.02±0.03	0.01±0.01	Bacteroidetes	Flavobacteriia	Flavobacteriales	Flavobacteriaceae	Flavobacterium	unclassified_Flavobacterium
	OTU1970	0.01±0.01	0±0	Bacteroidetes	Sphingobacteriia	Sphingobacteriales	unclassified_Sphingobacteriales	unclassified_Sphingobacteriales	unclassified_Sphingobacteriales
	OTU517	0.02±0.03	0.01±0.01	Chloroflexi	Ktedonobacteria	Ktedonobacterales	Ktedonobacteraceae	unclassified_Ktedonobacteraceae	unclassified_Ktedonobacteraceae
	OTU2860	0.01±0.01	0±0	Chloroflexi	Anaerolineae	SBR1031	oc28	unclassified_oc28	unclassified_oc28
	OTU1056	0.02±0.02	0.01±0.01	Elusimicrobia	Elusimicrobia	unclassified_Elusimicrobia	unclassified_Elusimicrobia	unclassified_Elusimicrobia	unclassified_Elusimicrobia
	OTU290	0.03±0.0	0.01±0.0	OD1	unclassified_OD1	unclassified_OD1	unclassified_OD1	unclassified_OD1	unclassified_OD1

		4	1						
	OTU713	0.01±0.01	0±0	Proteobacteria	Alphaproteobacteria	Rickettsiales	unclassified_Rickettsiales	unclassified_Rickettsiales	unclassified_Rickettsiales
	OTU1766	0±0.01	0±0	Proteobacteria	unclassified_Proteobacteria	unclassified_Proteobacteria	unclassified_Proteobacteria	unclassified_Proteobacteria	unclassified_Proteobacteria
	OTU970	0.01±0.01	0±0	Proteobacteria	Alphaproteobacteria	Rhodospirillales	unclassified_Rhodospirillales	unclassified_Rhodospirillales	unclassified_Rhodospirillales
	OTU1621	0.01±0.01	0±0	Proteobacteria	Gammaproteobacteria	Legionellales	Coxiellaceae	unclassified_Coxiellaceae	unclassified_Coxiellaceae
	OTU279	0.03±0.04	0.01±0.01	Proteobacteria	unclassified_Proteobacteria	unclassified_Proteobacteria	unclassified_Proteobacteria	unclassified_Proteobacteria	unclassified_Proteobacteria
	OTU1658	0±0.01	0±0	Proteobacteria	Alphaproteobacteria	unclassified_Alphaproteobacteria	unclassified_Alphaproteobacteria	unclassified_Alphaproteobacteria	unclassified_Alphaproteobacteria
	OTU3980	0±0	0±0	Proteobacteria	Deltaproteobacteria	Syntrophobacterales	Syntrophobacteraceae	unclassified_Syntrophobacteraceae	unclassified_Syntrophobacteraceae
	OTU3191	0.01±0.01	0±0.01	Proteobacteria	Gammaproteobacteria	Legionellales	Coxiellaceae	unclassified_Coxiellaceae	unclassified_Coxiellaceae
	OTU756	0.03±0.03	0.02±0.01	Proteobacteria	Deltaproteobacteria	Myxococcales	Polyangiaceae	unclassified_Polyangiaceae	unclassified_Polyangiaceae
	OTU457	0.07±0.07	0.03±0.03	Proteobacteria	Gammaproteobacteria	Legionellales	Coxiellaceae	Aquicella	unclassified_Aquicella
	OTU4827	0±0.01	0±0	TM6	SJA-4	unclassified_SJA-4	unclassified_SJA-4	unclassified_SJA-4	unclassified_SJA-4
	OTU686	0.02±0.03	0.01±0.01	TM7	TM7-1	unclassified_TM7-1	unclassified_TM7-1	unclassified_TM7-1	unclassified_TM7-1
	OTU2205	0±0.01	0±0	unclassified	unclassified	unclassified	unclassified	unclassified	unclassified
	OTU3622	0±0	0±0	unclassified	unclassified	unclassified	unclassified	unclassified	unclassified
	OTU3116	0.01±0.01	0±0	unclassified	unclassified	unclassified	unclassified	unclassified	unclassified
LoopSeq-W-module2（significantly enriched)	OTU5910	0.05±0.1	0.07±0.11	Proteobacteria	Alphaproteobacteria	Rhizobiales	Beijerinckiaceae	Roseiarcus	uncultured bacterium
	OTU21	0.1±0.26	0.05±0.06	Proteobacteria	Gammaproteobacteria	Betaproteobacteriales	Chromobacteriaceae	Pseudogulbenkiania	Ambiguous_taxa
	OTU3855	0.29±0.39	0.13±0.16	Firmicutes	Clostridia	Clostridiales	Clostridiaceae 1	Clostridium sensu stricto 8	uncultured bacterium
	OTU802	0.02±0.03	0.05±0.07	Firmicutes	Bacilli	Bacillales	Paenibacillaceae	Paenibacillus	Ambiguous_taxa
PacBio-W-module2（significantly enriched)	OTU287	0.07±0.08	0.04±0.04	Bacteroidetes	Chitinophagia	Chitinophagales	Chitinophagaceae	Flavisolibacter	Flavisolibacter_ginsengisoli
	OTU476	0.07±0.0	0.04±0.0	Bacteroidetes	Chitinophagia	Chitinophagales	Chitinophagaceae	Flavisolibacter	Flavisolibacter_rigui

		5	4						
	OTU20171	0.04±0.04	0.03±0.03	Firmicutes	Clostridia	Thermoanaerobacterales	Thermoanaerobacteraceae	Caldanaerobacter	Caldanaerobacter_subterraneus
	OTU682	0.04±0.05	0.02±0.03	Planctomycetes	Planctomycetia	Planctomycetales	Planctomycetaceae	Thermogutta	Thermogutta_terrifontis
	OTU21	0.13±0.09	0.07±0.06	Planctomycetes	Planctomycetia	Planctomycetales	Planctomycetaceae	Thermogutta	Thermogutta_terrifontis
	OTU1	0.39±0.2	0.26±0.19	Planctomycetes	Phycisphaerae	Phycisphaerales	Phycisphaeraceae	Algisphaera	Algisphaera_agarilytica
	OTU260	0.03±0.02	0.03±0.04	Proteobacteria	Alphaproteobacteria	Rickettsiales	Rickettsiaceae	Rickettsia	Rickettsia_bellii
PacBio-W-module3（significantly enriched)	OTU110	0.03±0.04	0.02±0.03	Chloroflexi	Ktedonobacteria	Ktedonobacterales	Thermosporotrichaceae	Thermosporothrix	Thermosporothrix_hazakensis
	OTU5038	0.09±0.09	0.05±0.07	Bacteroidetes	Chitinophagia	Chitinophagales	Chitinophagaceae	Niastella	Niastella_yeongjuensis
	OTU998	0.03±0.03	0.02±0.02	Planctomycetes	Planctomycetia	Planctomycetales	Isosphaeraceae	Singulisphaera	Singulisphaera_acidiphila
PacBio-W-module4（significantly enriched)	OTU2302	0.02±0.03	0.01±0.02	Bacteroidetes	Chitinophagia	Chitinophagales	Chitinophagaceae	Flavisolibacter	unclassified
	OTU5505	0.03±0.03	0.02±0.02	Chloroflexi	Anaerolineae	Anaerolineales	Anaerolineaceae	Anaerolinea	Anaerolinea_thermophila
	OTU434	0.05±0.04	0.02±0.02	Planctomycetes	Planctomycetia	Planctomycetales	Planctomycetaceae	Planctopirus	Planctopirus_limnophila
	OTU18405	0.02±0.02	0.01±0.02	Proteobacteria	Betaproteobacteria	Burkholderiales	Not_Available	unclassified	unclassified
	OTU8090	0.03±0.03	0.02±0.03	Proteobacteria	Betaproteobacteria	Burkholderiales	Burkholderiaceae	unclassified	unclassified
	OTU356	0.05±0.04	0.03±0.03	unclassified	unclassified	unclassified	unclassified	unclassified	unclassified
	OTU224	0.06±0.06	0.04±0.06	unclassified	unclassified	unclassified	unclassified	unclassified	unclassified
V4-W-module1（significantly deplete)	OTU2961	0±0	0.01±0.01	Verrucomicrobia	Pedosphaerae	Pedosphaerales	unclassified_Pedosphaerales	unclassified_Pedosphaerales	unclassified_Pedosphaerales
	OTU4499	0±0	0±0	TM7	SC3	unclassified_SC3	unclassified_SC3	unclassified_SC3	unclassified_SC3
	OTU1612	0±0.01	0.03±0.02	Proteobacteria	Deltaproteobacteria	Desulfuromonadales	Geobacteraceae	Geobacter	unclassified_Geobacter
	OTU191	0.06±0.07	0.39±0.39	Proteobacteria	Deltaproteobacteria	Desulfuromonadales	Geobacteraceae	Geobacter	unclassified_Geobacter
	OTU744	0±0.01	0.02±0.02	Proteobacteria	Deltaproteobacteria	Desulfuromonadales	Geobacteraceae	Geobacter	unclassified_Geobacter
	OTU243	0.01±0.0	0.06±0.0	Proteobacteria	Deltaproteobacteria	Desulfuromonadales	Geobacteraceae	Geobacter	unclassified_Geobacter

	1	1	6						
	OTU1327	0±0.01	0.02±0.02	Proteobacteria	Deltaproteobacteria	Desulfuromonadales	Geobacteraceae	Geobacter	unclassified_Geobacter
	OTU1020	0.01±0.01	0.05±0.04	Proteobacteria	Deltaproteobacteria	Desulfuromonadales	Geobacteraceae	Geobacter	unclassified_Geobacter
	OTU201	0.05±0.05	0.22±0.23	Proteobacteria	Deltaproteobacteria	Desulfuromonadales	Geobacteraceae	Geobacter	unclassified_Geobacter
	OTU354	0.02±0.03	0.09±0.1	Proteobacteria	Deltaproteobacteria	Desulfuromonadales	Geobacteraceae	Geobacter	unclassified_Geobacter
	OTU829	0.01±0.01	0.02±0.02	Proteobacteria	Alphaproteobacteria	Rhodospirillales	Acetobacteraceae	unclassified_Acetobacteraceae	unclassified_Acetobacteraceae
	OTU1329	0±0	0.01±0.01	Proteobacteria	Deltaproteobacteria	MBNT15	unclassified_MBNT15	unclassified_MBNT15	unclassified_MBNT15
	OTU247	0.03±0.05	0.09±0.1	Proteobacteria	Deltaproteobacteria	Desulfuromonadales	Geobacteraceae	Geobacter	unclassified_Geobacter
	OTU6754	0±0	0±0	Proteobacteria	Alphaproteobacteria	Rhodospirillales	Rhodospirillaceae	unclassified_Rhodospirillaceae	unclassified_Rhodospirillaceae
	OTU608	0.02±0.02	0.04±0.04	Proteobacteria	Deltaproteobacteria	Desulfobacterales	Desulfobulbaceae	Desulfobulbus	unclassified_Desulfobulbus
	OTU525	0.01±0.02	0.03±0.04	Proteobacteria	Betaproteobacteria	Methylophilales	Methylophilaceae	unclassified_Methylophilaceae	unclassified_Methylophilaceae
	OTU715	0.02±0.02	0.03±0.04	Proteobacteria	Betaproteobacteria	Rhodocyclales	Rhodocyclaceae	Uliginosibacterium	unclassified_Uliginosibacterium
	OTU1383	0±0	0.01±0.01	Elusimicrobia	Elusimicrobia	Elusimicrobiales	unclassified_Elusimicrobiales	unclassified_Elusimicrobiales	unclassified_Elusimicrobiales
	OTU568	0.01±0.01	0.03±0.02	Bacteroidetes	Bacteroidia	Bacteroidales	unclassified_Bacteroidales	unclassified_Bacteroidales	unclassified_Bacteroidales
	OTU598	0±0.01	0.03±0.03	Acidobacteria	Holophagae	Holophagales	Holophagaceae	unclassified_Holophagaceae	unclassified_Holophagaceae
	OTU3549	0±0	0.01±0.01	Acidobacteria	Solibacteres	Solibacterales	unclassified_Solibacterales	unclassified_Solibacterales	unclassified_Solibacterales
	OTU849	0.01±0.01	0.03±0.03	Acidobacteria	Holophagae	Holophagales	Holophagaceae	Geothrix	unclassified_Geothrix
	OTU813	0.02±0.02	0.05±0.03	Acidobacteria	Acidobacteriia	Acidobacteriales	Koribacteraceae	Candidatus_Koribacter	unclassified_Candidatus_Koribacter
LoopSeq-W-module2（significantly deplete)	OTU1516	0.07±0.11	0.09±0.12	Actinobacteria	Thermoleophilia	Solirubrobacterales	Solirubrobacteraceae	Solirubrobacter	Ambiguous_taxa
	OTU3580	0.1±0.18	0.13±0.13	Acidobacteria	Subgroup 5	uncultured bacterium	uncultured bacterium	uncultured bacterium	uncultured bacterium
LoopSeq-W-module1（significantly deplete)	OTU3367	0±0	0±0	Actinobacteria	Actinobacteria	Micrococcales	Microbacteriaceae	Amnibacterium	Amnibacterium kyonggiense
	OTU225	0±0	0±0	Actinobacteria	Actinobacteria	Corynebacteriales	Mycobacteriaceae	Mycobacterium	Mycobacterium sp. 'Bavariae'

	9								
PacBio-W-module1（significantly deplete)	OTU160	0.13±0.14	0.18±0.22	Bacteroidetes	Chitinophagia	Chitinophagales	Chitinophagaceae	Terrimonas	Terrimonas_pekingensis
	OTU879	0.02±0.03	0.05±0.06	Bacteroidetes	Chitinophagia	Chitinophagales	Chitinophagaceae	Terrimonas	Terrimonas_pekingensis
	OTU28	0.1±0.06	0.16±0.11	Planctomycetes	Planctomycetia	Planctomycetales	Planctomycetaceae	Gimesia	Gimesia_maris

Table S5 Key species of V4, LoopSeq and PacBio sequencing.

	OTU ID	Phylum	Class	Order	Family	Geuns	Species	Module	degree	closnesscentrality	betweenesscentrality
V4	OTU7307	Bacteroidetes	Sphingobacteriia	Sphingobacteriales	unclassified_Sphingobacteriales	unclassified_Sphingobacteriales	unclassified_Sphingobacteriales	1	768	0.658986	2908.464368
	OTU405	Verrucomicrobia	unclassified_Verrucomicrobia	unclassified_Verrucomicrobia	unclassified_Verrucomicrobia	unclassified_Verrucomicrobia	unclassified_Verrucomicrobia	2	760	0.657774	3628.870182
	OTU2287	Acidobacteria	DA052	Ellin6513	unclassified_Ellin6513	unclassified_Ellin6513	unclassified_Ellin6513	1	760	0.658379	3207.267699
	OTU769	Chlorobi	SJA-28	unclassified_SJA-28	unclassified_SJA-28	unclassified_SJA-28	unclassified_SJA-28	1	760	0.660203	4103.367426
LoopSeq	OTU1516	Actinobacteria	Thermoleophilia	Solirubrobacterales	Solirubrobacteraceae	Solirubrobacter	Ambiguous_taxa	2	50	0.627451	586.956944
	OTU5896	Actinobacteria	Thermoleophilia	Solirubrobacterales	Solirubrobacteraceae	Conexibacter	uncultured bacterium	2	50	0.623377	550.216189
	OTU791	Proteobacteria	Deltaproteobacteria	Desulfuromonadales	Geobacteraceae	Geobacter	uncultured bacterium	2	17	0.461538	63.215271
	OTU3146	Proteobacteria	Gammaproteobacteria	Betaproteobacteriales	Burkholderiaceae	Massilia	uncultured bacterium	2	45	0.413793	96.643262
PacBio	OTU17	Gemmatimonadetes	Gemmatimonadetes	Gemmatimonadales	Gemmatimonadaceae	Gemmatimonas	Gemmatimonas_phototrophica	3	79	0.646091	278.593105
	OTU18	Bacteroidetes	Chitinophagia	Chitinophagales	Chitinophagaceae	Flavisolibacter	unclassified	2	74	0.628	318.66482
	OTU355	Planctomycetes	Planctomycetia	Planctomycetales	Isosphaeraceae	Aquisphaera	Aquisphaera_giovannonii	2	72	0.623016	244.487707
	OTU10711	Planctomycetes	Phycisphaerae	Phycisphaerales	Phycisphaeraceae	Algisphaera	Algisphaera_agarilytica	4	72	0.625498	202.637967
